# A crucial exosome-related gene pair (*AAMP* and *ABAT*) is associated with inflammatory cells in intervertebral disc degeneration

**DOI:** 10.3389/fimmu.2023.1160801

**Published:** 2023-04-14

**Authors:** Huiyong Ren, Yumin Li, Hao Liu, Jiaxin Fan, Jie Li, Haopeng Li, Hongyu Wei, Liesu Meng, Shuai Cao

**Affiliations:** ^1^ Department of Orthopedics, Civil Aviation General Hospital, Beijing, China; ^2^ Department of Orthopaedics, The Second Affiliated Hospital of Xi’an Jiaotong University, Xi’an, Shanxi, China; ^3^ Department of Neurology, The Second Affiliated Hospital of Xi’an Jiaotong University, Xi’an, China; ^4^ Department of Orthopaedic Surgery, China-Japan Friendship Hospital, Beijing, China; ^5^ Institute of Molecular and Translational Medicine (IMTM), and Department of Biochemistry and Molecular Biology, School of Basic Medical Sciences, Xi’an Jiaotong University Health Science Center, Xi’an, China; ^6^ National Joint Engineering Research Center of Biodiagnostics and Biotherapy, Second Affiliated Hospital, Xi’an Jiaotong University, Xi’an, Shaanxi, China

**Keywords:** Intervertebral disc degeneration, exosomes, machine learning, gene pairs, immune infiltration

## Abstract

Identification of exosome-related genes (ERGs) and competing endogenous RNAs (ceRNAs) associated with intervertebral disc degeneration (IDD) may improve its diagnosis and reveal its underlying mechanisms. We downloaded 49 samples from Gene Expression Omnibus and identified candidate ERGs using differentially expressed ERGs (De-ERGs), exosome-related gene pairs (ERGPs), and machine learning algorithms [least absolute shrinkage and selection operator (LASSO) and support vector machine (SVM)]. Immune cell-related ERGs were selected *via* immune-infiltration analysis, and clinical values were assessed using receiver operating characteristic curves. Based on the De-ERGs, a ceRNA network comprising 1,512 links and 330 nodes was constructed and primarily related to signal transduction pathways, apoptosis-related biological processes, and multiple kinase-related molecular functions. In total, two crucial De-ERGs [angio-associated migratory cell protein (AAMP) and 4-aminobutyrate aminotransferase (ABAT)] were screened from results in De-ERGs, ERGPs, LASSO, and SVM. Increased *AAMP* expression and decreased *ABAT* expression were positively and negatively correlated with CD8^+^ T cell infiltration, respectively. *AAMP*/*ABAT* was the only pair differentially expressed in IDD and correlated with CD8^+^ T cell infiltration. Furthermore, *AAMP*/*ABAT* displayed higher accuracy in predicting IDD than individual genes. These results demonstrated the ERGP *AAMP*/*ABAT* as a robust signature for identifying IDD and associated with increased CD8^+^ T cell infiltration, suggesting it as a promising IDD biomarker.

## 1 Introduction

Intervertebral disc degeneration (IDD) is the main cause of low back pain and can result in work incapacity and a heavy socioeconomic burden (1). In the United States, the annual cost of low back pain is ~100 to 200 billion dollars (2). For the previous 200 years, IDD has been considered to be related to aging, excessive physical labor, and recently, to genetic factors (3, 4). Although various factors, such as age, genetics, and mechanical load, reportedly influence low back pain etiology, genetics is the crucial risk factor for IDD, accounting for ~70% of cases (1, 5). However, the genomic mechanisms of IDD have not been completely elucidated. Moreover, in the previous decade, extensive efforts have been made to discover novel treatments for the pathophysiological or genomic mechanisms of IDD, including through genome engineering, disc cell therapy, and the application of mesenchymal stem cells (MSCs) (1, 6–8). Few of these strategies have shown significant benefits in clinical practice; however, as research progresses, an increasing number of problems remain to be resolved (9–11).

Exosomes are vesicles (40–100 nm in size) with a membranous structure. Exosomes are released by cells and regulate communication within/between cells by delivering functional molecules, such as proteins, lipids, and nucleic acids (12). In recent years, exosomes as a novel cell-free treatment strategy showed potent effects with some advantages and have thus have been increasingly studied in the field of IDD. Additionally, MSC-driven exosomes may be the main or the only driver of MSC regeneration; therefore, they may represent a potential alternative treatment for IDD. Moreover, evidence suggests that exosomes are involved in many normal physiological processes, such as coagulation (13), autophagy, reproduction (14), and nervous system adjustment. Exosomes also exert important effects on pathological processes, such as degenerative diseases (15). For example, exosomes harvested from nucleus pulposus (NP) tissue derived from IDD patients can promote MSC migration into an NP-like phenotype (16) through the NOTCH1 pathway (17). Furthermore, exosomes may help prevent apoptosis (17) and promote the proliferation of NP cells (15). Importantly, use of exosomes as a cell-free therapy may circumvent many of the challenges associated with MSCs (13, 18, 19). Therefore, exosomes show considerable promise for potential applications in IDD therapy; however, exosome-related genes (ERGs) and their regulatory mechanisms remain unknown. Therefore, studies of exosomes in the context of IDD are needed to elucidate the molecular mechanisms of pathogenesis and provide insight to promote the development of treatment strategies.

This study identified and annotated important ERGs in IDD. We employed high-throughput data analysis and machine learning algorithms to analyze the expression profiles of IDD-related genes from public databases. The results provide a theoretical basis for the functions and molecular mechanisms of ERGs involved in IDD occurrence and/or development and may help the identification of new candidates for IDD gene therapy. Additionally, we screened ERGs with significantly different expression levels in order to explore their potential as diagnostic biomarkers for IDD.

## 2 Materials and methods

### 2.1 Data sourcing

The GSE70362, GSE56081, and GSE15227 datasets were collected from the National Center of Biotechnology Information Gene Expression Omnibus (GEO) database. The GSE70362 dataset includes 14 IDDs and 10 control samples, and the GSE50681 dataset comprises 5 IDDs and 5 control samples (details for the GEO datasets are shown in **Supplementary Table S1**). The GSE70362 and GSE56081 datasets were merged for subsequent analysis after removing batch effects in order to obtain standardized data using the combat function of the “SVA” package (Leek JT (2021). sva: Surrogate Variable Analysis. (Version 3.40.0), https://github.com/antass/sva). Based on the grouping basis of the two original datasets and the Thompson grading system, grades I through III were set as a control cohort, and grades IV through V were set as an IDD cohort (20, 21). To explore the role of ERGs in IDD, a comprehensive list of ERGs was obtained from the exoRbase database (http://www.exorbase.org/). To identify ERGs that are potentially relevant to IDD pathology, tissue-specific ERGs not expressed in the intervertebral disc tissue were excluded, resulting in a set of ERGs that possibly relate to IDD. We then used the “idmap3” package (Zeng JM (2023). idmap3: get the gene symbols of probes by hisat2 and gtf. (Version 0.1.0), https://github.com/jmzeng1314/idmap3) to annotate the merged datasets of GSE70362 and GSE56081 to screen ERGs expressed in the metadata by calculating the intersection of gene names and ERG lists. GSE15227 as an external validation dataset. Because the data were publicly collected from the GEO database, the release guidelines approved by the database were strictly followed.

### 2.2 Identification of differently expressed ERGs (De-ERGs)

The “limma” package (Smyth G (2021). Linear Models for Microarray Data Description: Data analysis, linear models and differential expression for microarray data. (Version 3.48.3), http://bioinf.wehi.edu.au/limma) was used for differential expression (DE) analysis between the IDD and control cohorts in the merged dataset. Heatmaps were generated to assess De-ERGs using the “pheatmap” package (Kolde R (2019). pheatmap: Pretty Heatmaps. (Version 1.0.12), https://CRAN.R-project.org/package=pheatmap). The De-ERG cut-off values were set as a fold-change (FC) >1.5 with a *p* < 0.05 (adjusted).

### 2.3 Construction of a competing endogenous RNA (ceRNA) network of De-ERGs

A ceRNA network was derived from differentially expressed long noncoding RNAs (De-lncRNAs), micro (mi)RNAs, and De-ERGs to reveal the mechanism of interaction between RNAs that regulate ERG expression. The GSE56081 dataset included the lncRNA expression profile of human IDD. The “limma” package was used to perform DE analysis of lncRNAs among IDD and control cohorts. Downstream miRNAs were then predicted by the De-lncRNAs through the miRcode database (highly conservative). Furthermore, the miRDB, miRTarBase, and TargetScan databases were used to identify downstream mRNA targets (at least two databases predicted) of the above miRNAs. Finally, the ceRNA network of De-ERGs was constructed with the De-lncRNAs, miRNAs, mRNA targets, and De-ERGs obtained as described and visualized using Cytoscape (v.3.8.0; https://cytoscape.org/) (**Supplementary Table S2** lists the addresses for all of the databases).

### 2.4 Functional annotations

Gene Ontology (GO) functional annotation and Kyoto Encyclopedia of Genes and Genomes (KEGG) pathway annotation analyses can define terms linked with gene function and the interrelationships among the De-ERGs. GO and KEGG pathway analyses were performed to annotate and analyze the biological pathways and functions of the obtained De-ERGs *via* the “clusterProfiler” (Wu T (2021). clusterProfiler 4.0: A universal enrichment tool for interpreting omics data. (Version 4.0.5), https://github.com/GuangchuangYu/clusterProfiler), “org.Hs.eg.db” (Carlson M (2021). org.Hs.eg.db: Genome wide annotation for Human. (Version 3.13.0)) and “DOSE” (Yu GC (2015). DOSE: Disease Ontology Semantic and Enrichment analysis. (Version 3.18.3), https://github.com/GuangchuangYu/DOSE) packages. The “pheatmap” package was used to visualize GO and KEGG results as dot plots (*p* < 0.05 and Q < 0.05).

### 2.5 Establishment of signature ERG pairs (ERGPs)

We established a list of ERGPs for subsequent analysis according to the recommended gene pair method, which can ignore the batch effect when merging GSE70362 and GSE56081 datasets for constructing ERGPs. The gene pair method suggests that if the expression level of the ERG is higher than the latter ERG expression level in a specific and formed ERGP, the score of this ERGP is 1; otherwise, the ERGP score is 0. We screened all ERGPs in the merged datasets and then generated a heatmap using the “pheatmap” package in order to display differences in ERGP expression.

### 2.6 Screening of ERG features based on machine learning

The least absolute shrinkage and selection operator (LASSO), a machine learning algorithm, uses regularization to improve prediction accuracy. The support vector machine (SVM) is a generalized linear classifier that performs binary classification of data according to supervised machine learning. To identify the significant genes associated with the difference between IDD and control groups, we used LASSO and SVM to identify crucial feature genes. The Venn method was used to find the intersection of crucial feature genes obtained from LASSO, those from SVM, genes in ERGPs, and De-ERGs. The overlapping ERG features from the four sets were then used as candidate exosome-related markers for IDD. Based on the expression levels of the ERGs in the merged dataset, the SVM was employed using the “caret” package (Kuhn M (2022). caret: Classification and Regression Training. (Version 6.0-93), https://topepo.github.io/caret) with five-fold cross (5×) validation to screen crucial ERGs. Moreover, the random seed was set to 85 for both LASSO and SVM employment. Machine learning with 47 ERGPs and De-ERGPs was used to identify crucial genes based on the Venn method. The expression levels of candidate markers in the merged dataset (training set) were determined to verify the accuracy of the feature gene selection. Furthermore, the expression level of candidate makers in the GSE15227 dataset (validation set) was measured to verify the extrapolation ability. GraphPad Prism (v.8.0; GraphPad Software, La Jolla, CA, USA) was used to visualize the results by generating a histogram of crucial ERG expression levels.

### 2.7 Immune cell-infiltration and correlation analyses among crucial ERGs and infiltrated immune cells

We used a novel computational method (the CIBERSORT algorithm) to analyze different immune cell types and estimate the enrichment of various immunocyte compositions of tissues using 547 reference gene-expression values. We used CIBERSORT to detect the heterogeneities or types of infiltrated immune cells in the IDD and control cohorts (1000 permutations) and obtained a matrix of 22 types of infiltrating immune cells and a correlation file for the immune cells. A CIBERSORT *p* < 0.05 was set as a critical value. Various R packages were used to visualize the results for different purposes. The radar chart was created with the “fmsb” package (Nakazawa M (2023). fmsb: Functions for Medical Statistics Book with some Demographic Data. (Version 0.7.5), https://CRAN.R-project.org/package=fmsb) to show the difference in infiltrating CD4+ and CD8+ T cells and their subtypes between the IDD and control cohorts. The correlation map was generated with the “corrplot” package (Wei TY, Simko V (2021). R package ‘corrplot’: Visualization of a Correlation Matrix. (Version 0.92), https://github.com/taiyun/corrplot.&quot). Furthermore, correlation analysis (Spearman’s rank) was conducted to analyze the correlation between crucial ERG-expression levels and infiltrating immune cells, and the “ggplot2” package (Wickham H (2016). ggplot2: Elegant Graphics for Data Analysis. (Version 3.4.1), https://ggplot2.tidyverse.org) was used for visualization.

### 2.8 Assessment of the effectiveness of candidate ERG biomarkers

After screening the crucial ERGs and ERGPs, we used a receiver operating characteristic (ROC) curve to assess the effectiveness of the expression data from the merged datasets to test the discriminating ability of the identified biomarkers between the IDD and control cohorts. The “survivalROC” package (Heagerty PJ (2022). survivalROC: Time-Dependent ROC Curve Estimation from Censored Survival Data. (Version 1.0.3.1), https://github.com/taiyun/corrplot) was used for ROC analysis to assess the area under the ROC curve (AUC) value. Furthermore, we verified the value of these biomarkers using the GSE15227 dataset.

### 2.9 Statistical analyses

All statistical analyses and construction of the graphics were performed using R software (v.4.0.5; https://www.r-project.org/) and GraphPad Prism (v.8.0; GraphPad Software). The critical selection value adopted for DE analysis was *p* < 0.05 and FC >1.5. ROC curves with their AUC values were used to assess the performance and effectiveness of the candidate ERG biomarkers. A *p-value* < 0.05 was considered statistically significant.

## 3 Results

### 3.1 Data preprocessing

**Figure 1:**
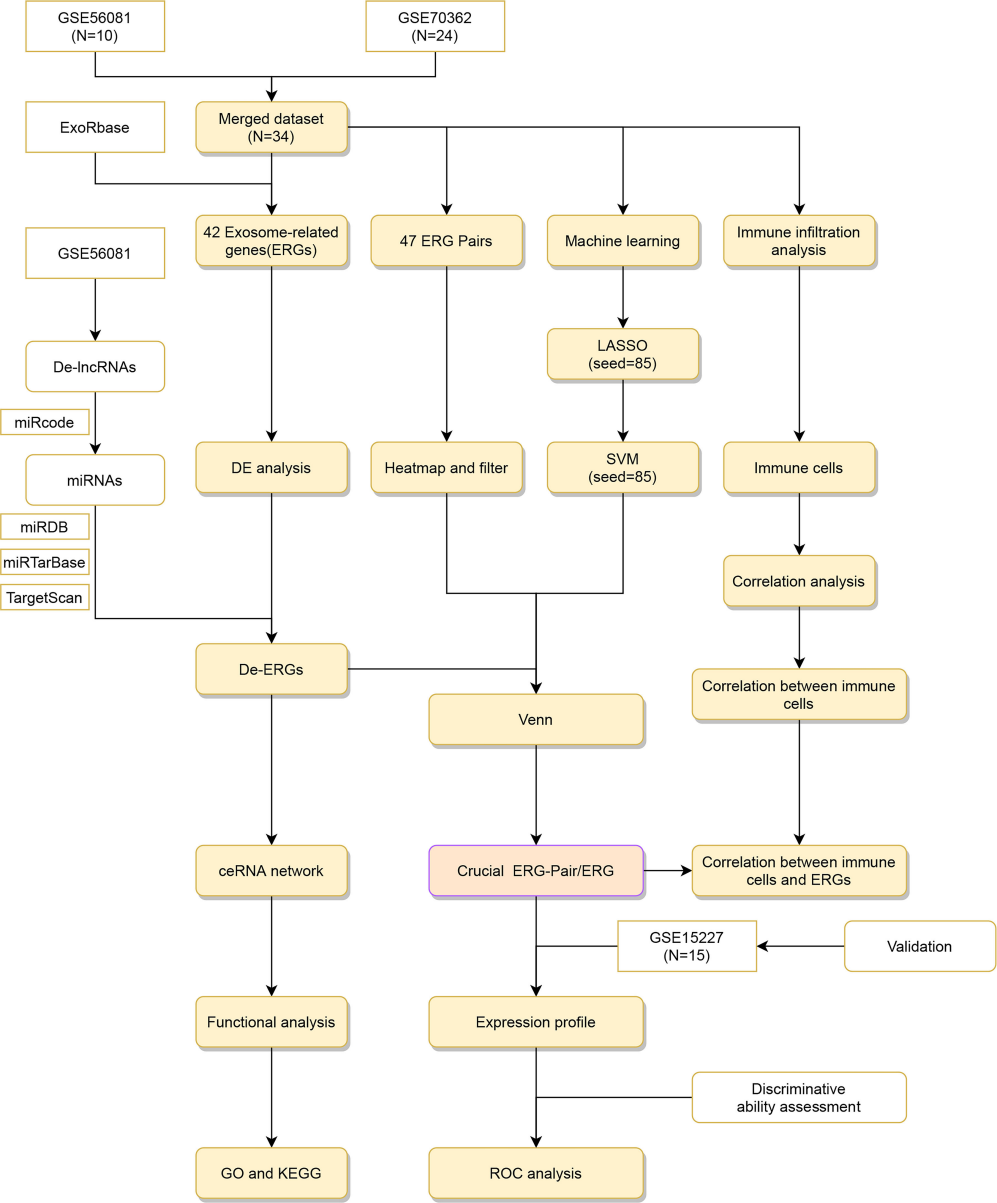
Flow diagram. ERG, Exosome-related genes.

After normalization, we merged the expression data of the GSE70362 and GSE56081 datasets. A total of 179 ERGs were collected from the exoRbase database, and after removing 31 tissue-specific ERGs, 148 non-tissue-specific ERGs, potentially associated with IDD, were obtained (**Supplementary Table S3**). Using the Venn method, we identified 42 ERGs for subsequent analysis at the intersection of the merged dataset and the 148 ERGs. The design and process of this study are shown in **Figure 1**.

### 3.2 Identification of De-ERGs in IDD

**Figure 2:**
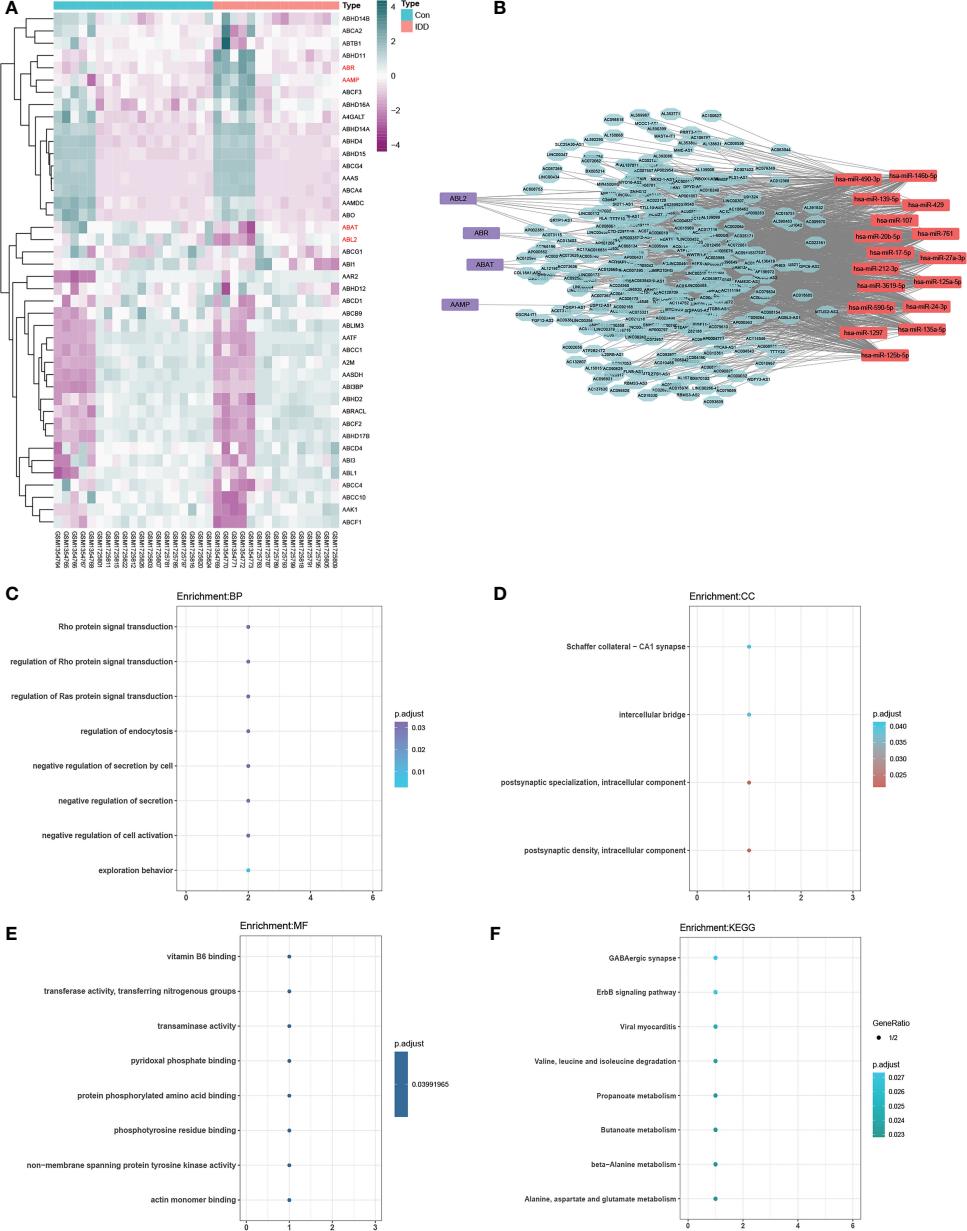
**(A)** Heat map of exosome-related genes (ERGs). The ceRNA network, including 330 lncRNAs, 17 miRNAs, and four differentially expressed ERGs (*p*<0.05) **(B)**, purple represents differentially expressed ERGs), is functionally annotated by GO (biological process, **(C)**; cellular component, **(D)**; molecular function, **(E)** and KEGG **(F)**. Filter conditions are adjusted *p*-value < 0.05 and q value < 0.05. Details are shown in **Supplementary Tables S4** and **S5**.

We identified four De-ERGs in the merged dataset with DE analysis. The log_2_FC showed that (4-aminobutyrate aminotransferase) *ABAT* and (ABL proto-oncogene 2, non-receptor tyrosine kinase) *ABL2* were downregulated, whereas angio-associated migratory cell protein (*AAMP*) and active breakpoint cluster region-related protein (*ABR*) were upregulated. The heatmap in **Figure 2A** shows the DE of ERGs between the IDD and control groups, as well as the four De-ERGs with FC >1.5 and *p <*0.05.

### 3.3 ceRNA network construction

The source for ceRNA network construction was De-lncRNAs, miRNAs that interact with De-lncRNAs, and target mRNAs (subsequently intersecting with De-ERGs). We detected a total of 330 De-lncRNAs in the GSE56081 dataset with DE analysis, and 17 miRNAs were predicted to interact with these De-lncRNAs according to the miRcode database. Furthermore, 17 downstream target mRNAs related to the 17 miRNAs were predicted using the three public databases mentioned in section 2.3. Four ERGs (*ABAT, ABL2, AAMP*, and *ABR*) were revealed as overlapping mRNAs between the 17 target mRNAs and De-ERGs. Therefore, we constructed the ceRNA network (**Figure 2B**) using the De-lncRNAs, miRNAs, and four overlapping De-ERGs to reveal the underlying molecular mechanism of IDD. The ERG-related ceRNA network contained 1,512 edges and 351 nodes, indicating that these ceRNAs are involved in regulating the expression of the four De-ERGs.

### 3.4 Functional enrichment analyses

GO enrichment analysis for the biological process category (**Figure 2C**; **Supplementary Table S4**) revealed that the ERG-related ceRNA network was mainly enriched in exploration behavior (GO:0035640), regulation of the Rho protein signal (GO:0035023), negative cell activation regulation (GO:0050866), Rho protein signal transduction (GO:0007266), negative regulation of secretion (GO:0051048), and regulation of endocytosis (GO:0030100). **Figures 2D, E** show the GO enrichment results for the cellular component and molecular function categories, respectively. KEGG enrichment analysis (**Figure 2F**; **Supplementary Table S5**) revealed enriched pathways of butanoate metabolism (hsa00650); beta-alanine metabolism (hsa00410); propanoate metabolism (hsa00640); alanine, aspartate, and glutamate metabolism (hsa00250); and ErbB signaling pathway (hsa04012). **Tables S4** and **S5** list the GO and KEGG analysis results in more detail. Functional enrichment analysis showed that the ERG-related ceRNA network was mainly related to GABA metabolism, regulation of the Rho family, and endocytosis regulation.

### 3.5 Construction of an ERGP signature

**Figure 3:**
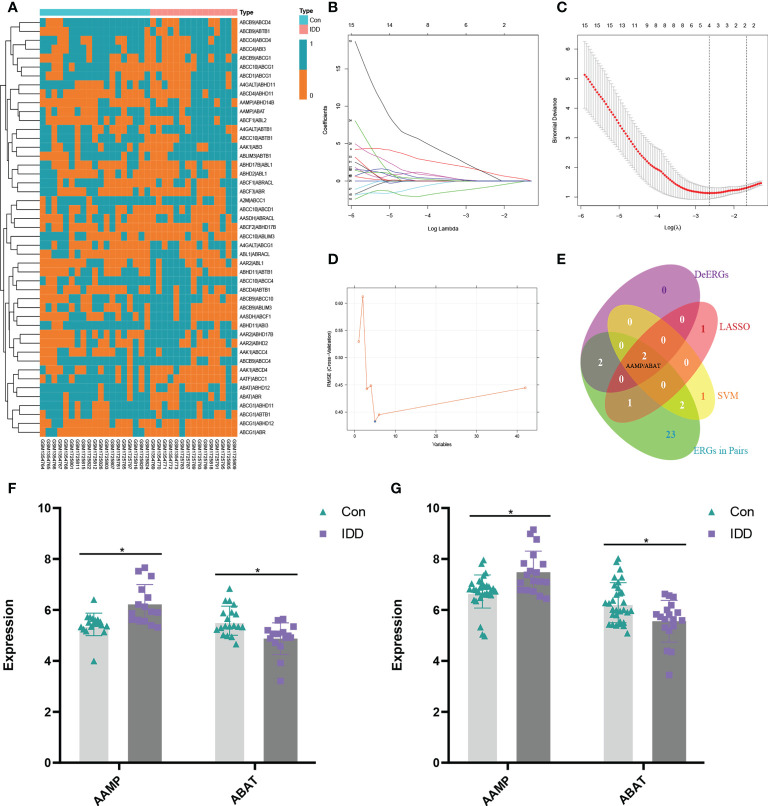
**(A)** Heat map of exosome-related gene pairs (ERGPs). Based on the expression level of 42 exosome-related genes (ERGs), the machine learning methods, including LASSO regression **(B, C)**; seed=85) and SVM **(D)** (seed=85), were used to screen crucial ERGs. The Venn method is used to screen for candidate crucial ERG symbol(s) in differentially expressed ERGs (De-ERGs), LASSO, SVM, and ERGPs **(E)**. Finally, the Venn diagram **(E)** is shown that two genes (*AAMP* and *ABAT*) are crucial ERGs and construct the crucial ERGP (*AAMP*/*ABAT*). The expression profile for *AAMP* and *ABAT* **(F)**. The expression profile for *AAMP* (upregulation) and *ABAT* (downregulation) in the validation set, which merged with GSE15227 **(G)**. *, *p* < 0.05.

Using the Venn method, we obtained 148 ERGs from the exoRbase database and 42 ERGs in the merged dataset. We subsequently generated 47 ERGPs comprising 30 ERGs using pairwise gene calculations. **Figure 3A** shows the heatmap of 47 ERGPs between the IDD and control groups.

### 3.6 Identification and validation of the feature biomarkers

We identified potential ERG biomarkers using the SVM and LASSO algorithms. The LASSO algorithm was used to identify four candidate biomarkers from the 42 ERGs for IDD: *AAMP, ABAT, ATP-binding cassette sub-family D member 4*, and *abhydrolase domain-containing 16A phospholipase* (**Figures 3B, C**). The SVM algorithm was used to screen a subset of five ERGs as biomarkers: *AAMP, ABAT, AAR2 splicing factor*, *aminoadipate-semialdehyde dehydrogenase*, and *ABI-binding protein 1* (**Figure 3D**). The two overlapping ERGs (*AAMP* and *ABAT*) obtained with the Venn method from LASSO (4 ERGs) and SVM (5 ERGs) and the four De-ERGs and 30 ERGs among the ERGPs were considered candidate ERG biomarkers for subsequent analysis (**Figure 3E**). **Figure 3F** shows the expression levels of *AAMP* and *AABT* as determined using the metadata of the merged dataset (merged with GSE15227), which was further used as a validation dataset to verify the expression profiles of the two ERGs in order to ensure the authenticity of the results. The expression level of *AAMP* in IDD NP tissue was significantly higher than that in the control, whereas that of *ABAT* in IDD NP tissue was significantly lower than that in the control (**Figure 3G**) (*p* < 0.05). Therefore, *AAMP, ABAT*, and the ERGP comprising *AAMP* and *ABAT* (*AAMP/ABAT*) were considered as potentially reliable biomarkers.

### 3.7 Immune cell infiltration

**Figure 4:**
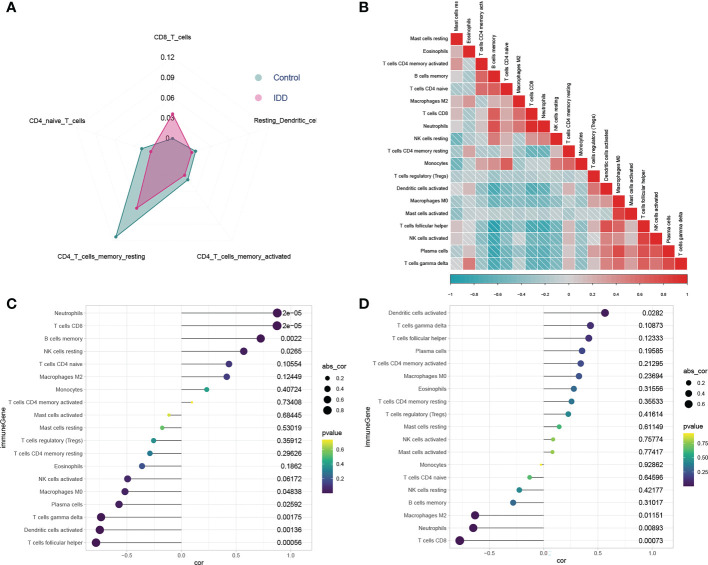
Analysis of immune cell infiltration and the relationship between exosome-related gene (ERG) and immune cells. Radar chart of immune infiltration between control and IDD groups **(A)**. A correlation heat map is used to show the correlation between Infiltrated immune cells **(B)**. The correlation diagram is used to show the relationship between infiltrated immune cells and the crucial ERGs **(C, D)**. The increased CD8^+^ T cells infiltration is positively correlated with highly expressed *AAMP* **(C)**, whereas it is negatively correlated with the low expressed *ABAT* **(D)**. The Calculation results between ERGs and immune cells are presented in more detail in **Supplementary Table S6**.

Differences in the proportions of infiltrated immune cells in the NP tissue between IDD and control samples are shown in **Figure 4A**. The proportions of resting CD4^+^ memory T cells and resting dendritic cells in IDD NP tissues were statistically lower than those in the control cohort. By contrast, the population of CD8^+^ T cells was significantly higher in IDD NP tissues than in normal tissues. These findings suggested that during IDD development, the immune cell-infiltration profile in NP tissue changed, indicating that the pathological process of IDD involves complex regulatory mechanisms associated with immune cell infiltration. **Figure 4B** shows a heatmap representing the correlation between infiltrated immune cells in samples of the merged dataset.

### 3.8 Correlation between AAMP/ABAT and immune cells

Analysis of infiltrated immune cells in NP tissue revealed their potential roles in IDD pathogenesis. Therefore, we investigated possible correlations between the level of infiltrated immune cells in NP tissue and ERG expression. Correlation analysis suggested that *AAMP* expression (**Figure 4C**) mainly showed a significantly positive correlation with the CD8^+^ T cell (*p* < 0.001), neutrophil (*p* < 0.001), and memory B-cell (*p* = 0.002) populations and a negative correlation with populations of activated dendritic cells (*p* = 0.001), follicular helper T cells (*p* < 0.001), plasma cells (*p* = 0.026), and macrophages (M0) (*p* = 0.048). Conversely, *ABAT* expression (**Figure 4D**) was mainly positively correlated with populations of resting dendritic cells (*p* = 0.0282) and negatively correlated with those of CD8^+^ T cells (*p* < 0.001), neutrophils (*p* = 0.009), and M2 macrophages (*p* = 0.012). Interestingly, an increase in CD8^+^ T cell infiltration was positively correlated with increased *AAMP* expression but negatively correlated with decreased *ABAT* expression, which indicated that *AAMP* and *ABAT* contribute to the differences in CD8^+^ T cell infiltration. Additionally, the increased infiltration of activated dendritic cells was positively correlated with elevated *ABAT* expression but negatively correlated with decreased *AAMP* expression; however, this did not appear to result in a difference in the number of infiltrating dendritic cells between degenerative and control intervertebral discs.

### 3.9 Assessment of the efficacy of the biomarkers for distinguishing IDD

**Figure 5:**
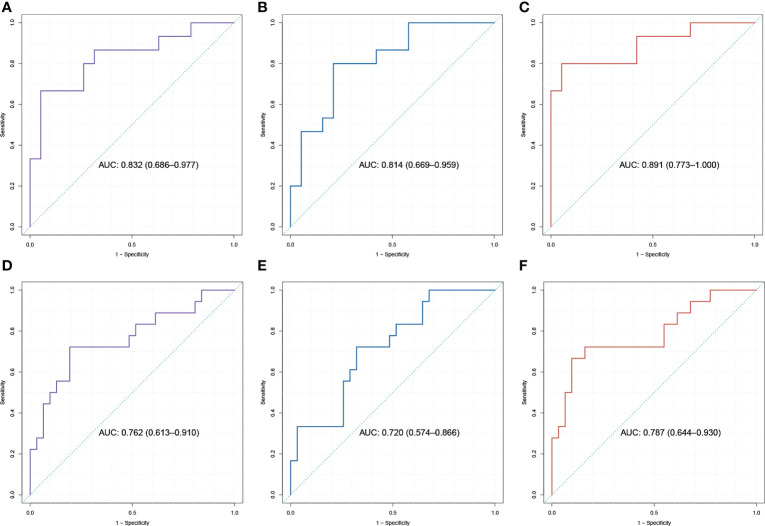
The ROC analysis for crucial exosome-related genes (ERGs) and ERG Pair (ERGP). In the training dataset **(A–C)** and validation dataset, which merged with GSE15227 **(D–F)**, the area under the curves (AUCs) of the ROCs is displayed that ROCs of ERGP **(C, F)** performs better than ROCs of *AAMP* **(A, D)** or *ABAT* **(B, E)** in distinguishing IDD.

We then generated a ROC curve to test the ability of *AAMP, ABAT*, and *AAMP/ABAT* to distinguish IDD. The AUC values were 0.832 [95% confidence interval (CI): 0.686–0.977; *p* < 0.001] for *AAMP*, 0.814 (95% CI: 0.669–0.959; *p* < 0.001) for *ABAT*, and 0.891 (95% CI: 0.773–1.00, *p* < 0.001) for *AAMP/ABAT* in the merged dataset (GSE70362 and GSE56081) (**Figures 5A–C**). The AUC values of the training cohort demonstrated the superior ability of the three biomarkers to distinguish IDD from control samples. Furthermore, we confirmed this discriminatory ability in the GSE15227 external verification set, with an AUC of 0.762 (95% CI: 0.613–0.900, *p* < 0.001) for *AAMP*, 0.702 (95% CI: 0.574–0.866, *p* = 0.003) for *ABAT*, and 0.787 (95% CI: 0.644–0.930, *p* < 0.001) for *AAMP/ABAT* (**Figures 5D–F**). Moreover, ERGP showed better performance in distinguishing IDD from control samples as compared with use of either *ABAT* or *AAMP*, and *AAMP/ABAT* also demonstrated potential as a diagnostic reference, with a specificity of 0.8 and sensitivity of 0.947 (**Supplementary Table S7**).

## 4 Discussion

There is currently no clear evidence that the harsh microenvironment of the intervertebral disc limits the response of cells to exosomes, showing their unique advantages in the treatment of IDD (22, 23). However, most studies on exosomes have focused on the field of oncology, and research on the regulation of exosomes in IDD is still relatively limited. Therefore, although exosomes show excellent therapeutic potential against IDD, their regulatory mechanisms remain unclear. In the present study, we employed machine learning algorithms to screen crucial ERGs potentially involved in IDD pathogenesis in order to gain insight for follow-up studies of exosomes in the context of IDD.

We found that *AAMP* expression was upregulated and *ABAT* downregulated in IDD NP tissues. AAMP, initially isolated from a melanoma cell line (24), is a pro-tumor protein that contributes to proliferation, angiogenesis, and other biological activities and can be secreted into the extracellular matrix (25, 26). Current research on AAMP mainly focuses on the mechanism of tumorigenesis, and there are no relevant reports on IDD. A previous study revealed that AAMP expression is more typical in activated T lymphocytes (26), and the present study showed that increased CD8^+^ T cell infiltration was significantly positively correlated with high expression of *AAMP*, which may recruit a significantly higher proportion of CD8^+^ T cells to IDD NP tissue. This suggests that AAMP may play a crucial role in IDD by affecting exosomal content and changing the immune cell-infiltration profile during IDD pathogenesis. Additionally, low expression of *ABAT* may also contribute to IDD, in that CD8^+^ T cell infiltration under that condition was significantly negatively correlated with IDD. These results suggest a link between the relationship between exosomes and immune cell infiltration in IDD. ABAT (GABA transaminase), which is located in mitochondria or neurons in humans, has been studied mainly in prokaryotes; therefore, its potential roles in mammalian systems remain poorly understood (27). Previous studies showed that ABAT-mediated GABA synthesis and catabolism are highly active during periods of nutrient deficiency and environmental stress, indicating its importance in cell-survival mechanisms (27, 28). However, there are no studies on the related mechanism of ABAT in IDD. As noted, the present findings suggest that ABAT may contribute to changes in immune cell infiltration during the process of IDD; however, additional studies are required to confirm these findings. ABL2, a non-receptor tyrosine kinase, could phosphate the following adaptors and result in a series of biological seffects, plays an important role along with ABL1 in processes linked to cell survival and growth, such as response to extracellular stimuli, cytoskeleton remodeling, and receptor endocytosis (29, 30). This suggests that the regulatory mechanism of ABL2 in IDD might be related to its regulation of the cell response to extracellular stimuli to promote the functions of IDD-derived exosomes. ABR belongs is a small GTPase of the Rho family, which plays an important role in regulating the biological behavior of macrophages by specifically regulating the functions of Rho family members (31, 32). GO annotation revealed that the four De-ERGs were mostly enriched in the regulation of Rho protein signal transduction and Ras protein signal transduction. Additionally, Ras and its homologous family member RhoA participate in the regulation of mechanically induced fibrosis in various organ systems. Importantly, Meng et al. (33) found that the RhoA/myocardin-related transcription factor A translocation pathway promotes mechanical overload-induced fibrogenic activity in NP tissue, suggesting that the ERGs identified in the present study might be involved in regulating the fibrotic process induced by mechanical forces in the intervertebral disc through the regulation of Rho family members.

KEGG pathway annotation indicated that the four De-ERGs were mainly enriched in the GABAergic synapse and ErbB signaling pathways. Previous studies revealed that ErbB signaling regulates cell proliferation, migration, differentiation, apoptosis, and motility by mediating the phosphoinositide 3-kinase/Akt, Janus kinase/signal transducer and activator of transcription, and mitogen-activated protein kinase signaling pathways, with activation of the latter pathway in IDD possibly leading to apoptosis of annulus fibrosus and NP cells (34, 35). Therefore, the ERGs identified in the present study may regulate NP cell apoptosis through the ErbB signaling pathway, which mediates other signaling pathways.

CeRNA networks demonstrate their potential roles in regulating the expression of various genes, as well as the pathogenesis of various diseases. Increasing evidence shows that miRNAs are involved in many aspects of cell function, including proliferation, apoptosis, and inflammation, and thereby modulate a series of pathophysiological changes that affect many processes (36). In the present study, we identified 330 De-lncRNAs and predicted 17 miRNAs that interact with them, resulting in construction of the ceRNA network. This network identified mir-125a-5p as targeting *ABL2*, with a previous study reporting that the miR-125a-5p/ABL2 axis plays a crucial role in suppressing the proliferation and migration of cervical carcinoma (37). Our present findings show that the most enriched pathway regulated by the ceRNA-AAMP/ABAT network is a biological process related to the positive regulation of phospholipase activity. ABL2 is the most significant DE phospho-proteins associated with this pathway, and downregulated ABL2 means the phospholipase activity is negatively regulated in IDD. These findings suggest that the identified De-ERGs that are regulated by ceRNAs may play a crucial role in IDD.

Immune cell infiltration of the intervertebral disc is controversial, with some researchers believing that the relatively closed microenvironment of the intervertebral disc precludes significant infiltration of immune cells from the circulation and limits their role in IDD. However, increasing evidence suggests that blood-derived immune cells infiltrate degenerated intervertebral discs in specific instances and promote the inflammatory response after the proinflammatory stage by secreting inflammatory cytokines, such as interferon-γ, interleukin (IL)-1α, IL-1β, IL-8, IL-10, and neurogenic factors (38). The present results support this process. Other studies showed that in herniated discs, there is infiltration of macrophages, CD4^+^ T cells, and CD8^+^ T cells along with invading blood vessels (39). The migration of immune cells to the intervertebral disc stimulates inflammation but is also accompanied by the appearance of nociceptive nerve fibers from the dorsal root ganglion and increased discogenic pain (40, 41). In the present study, infiltration of CD8^+^ T cells increased in the NP of the IDD cohort, whereas infiltration of resting CD4^+^ memory T cells and resting dendritic cells decreased. Moreover, the increased CD8^+^ T cell infiltration was positively correlated with elevated *AAMP* expression and negatively correlated with low *ABAT* expression. These results show that the expression of the ERGP *AAMP/ABAT* correlated with immune cell infiltration, especially CD8^+^ T cells, suggesting potential roles for *AAMP* and *ABAT* in the pathological process of IDD.

According to our clinical experience and previous studies, IDD misdiagnosis happens rarely but does occur. Therefore, identification of reliable biomarkers capable of both distinguishing IDD from other maladies and diagnosing IDD patients is needed. Compared with use of a single gene as a biomarker, the use of gene pairs demonstrates better performance in discriminating degenerative discs from a control group (42). Furthermore, a comparison between **Figure 3A** and **Figure 2A** reveals that utilizing gene pairs for data integration and batch effect removal yields significantly better results than conventional data merging methods. In the present study, we identified a novel diagnostic signature from 47 ERGPs of IDD samples and verified it using an external dataset. Based on AUC values of 0.891 (95% CI: 0.773–1.00, *p* < 0.001) for the training set and 0.787 (95% CI: 0.644–0.930, *p* < 0.001) for the verification set, the ERGP *AAMP/ABAT* showed excellent performance in identification IDD. The findings suggest that this ERGP represents a potentially effective biomarker to help distinguish between control and degenerated intervertebral discs. Moreover, differences in ERG expression in IDD may provide insights for future investigations of the mechanisms, pathogenesis, and treatment of IDD. The discovery of novel gene markers may potentially provide targets for future gene therapy, ultimately benefiting the general public.

This work has some limitations. Although we merged two datasets in order to increase the sample size, the results and reliability of these findings need to be confirmed using additional clinical samples or datasets, although more samples may not be readily obtainable under normal conditions. Furthermore, the retrospective nature of the study suggests that further experimental verifications, such as examination of RNAs in the ceRNA network, are needed to confirm these findings. And further evaluation is required as our study did not further consider the age-specific distinction of AAMP/ABAT for distinguishing IDD.In summary, we identified *AAMP, ABAT, ABR*, and *ABL2* as De-ERGs that were then combined with 330 lncRNAs and 17 miRNAs to form a ceRNA network that suggested their potential roles in IDD. Moreover, the use of machine learning algorithms identified AAMP and ABAT as potentially efficacious biomarkers for distinguishing degenerative discs from a control cohort. Additionally, the results suggested that *AAMP/AABT* may influence changes in the immune cell-infiltration profile during IDD pathogenesis by regulating the proportion of CD8^+^ T cells in IDD NP tissue.

## Data availability statement

The original contributions presented in the study are included in the article/**Supplementary Material**. Further inquiries can be directed to the corresponding authors.

## Author contributions

SC, LM and HW designed the experiments. All authors performed the experiments and acquired the data. SC and HR analyzed the data. SC and HR wrote the manuscript. HLiu, JL, HLi and YL supervised the project. All authors contributed to the article and approved the submitted version.
