# Supplementary material for: A crucial exosome-related gene pair (AAMP and ABAT) is associated with inflammatory cells in intervertebral disc degeneration
Source: Front Immunol. 2023 Apr 14;14:1160801. doi: 10.3389/fimmu.2023.1160801 (PMC10140513; doi:10.3389/fimmu.2023.1160801)
Supplement: Supplementary file 1 [file Table_1.docx]

Article

**A crucial exosome-related gene pair (*AAMP* and *ABAT*) is associated with inflammatory cells in intervertebral disc degeneration**

**Supplementary Table S1.** Details for studied GEO datasets.

| **Datasets** | **Set for** | **Groups** | **N** | **Platform** | **Access URL** |
| --- | --- | --- | --- | --- | --- |
| GSE116726 | miRNA |  |  | GPL20712 | https://www.ncbi.nlm.nih.gov/geo/query/acc.cgi?acc=GSE116726 |
|  |  | Control | 3 |  |  |
|  |  | IDD | 3 |  |  |
| GSE56081 | lncRNA & mRNA |  |  | GPL15314 | https://www.ncbi.nlm.nih.gov/geo/query/acc.cgi?acc=GSE56081 |
|  |  | Control | 5 |  |  |
|  |  | IDD | 5 |  |  |
| GSE70362 | mRNA |  |  | GPL17810 | https://www.ncbi.nlm.nih.gov/geo/query/acc.cgi?acc=GSE70362 |
|  |  | Control | 14 |  |  |
|  |  | IDD | 10 |  |  |
| GSE15227 | mRNA |  |  | GPL1352 | https://www.ncbi.nlm.nih.gov/geo/query/acc.cgi?acc=GSE15227 |
|  |  | Control | 12 |  |  |
|  |  | IDD | 3 |  |  |

**Abbreviations:** GEO: Gene Expression Omnibus. Platform infor., Platform information. IDD, Intervertebral disc degeneration.

**Supplementary Table S2.** URLs details for obtained databases.

| **Database** | **URLs** |
| --- | --- |
| GEO | https://www.ncbi.nlm.nih.gov/gds |
| exoRbase | http://exorbase.org/database/ |
| Mircode | http://www.mircode.org/ |
| miRDB | http://mirdb.org/ |
| TargetScan | http://www.targetscan.org/vert_72/ |

**Abbreviations:** GEO: Gene Expression Omnibus.

**Supplementary Table S3.** The 148 Exosome-related gene (ERG) symbols/The ERG gene set.

| A2M | A2ML1 | A3GALT2 | A4GALT | A4GNT | AAAS | AACS | AADAT |
| --- | --- | --- | --- | --- | --- | --- | --- |
| AAED1 | AAGAB | AAK1 | AAMDC | AAMP | AAR2 | AARS | AARS2 |
| AARSD1 | AASDH | AASDHPPT | AASS | AATF | AATK-AS1 | ABAT | ABC13-47488600E17.1 |
| ABC13-47656900H15.1 | ABC7-42404400C24.1 | ABCA1 | ABCA10 | ABCA13 | ABCA2 | ABCA4 | ABCA5 |
| ABCA6 | ABCA7 | ABCA8 | ABCA9 | ABCB10 | ABCB5 | ABCB6 | ABCB7 |
| ABCB8 | ABCB9 | ABCC1 | ABCC10 | ABCC4 | ABCC5 | ABCC9 | ABCD1 |
| ABCD2 | ABCD3 | ABCD4 | ABCE1 | ABCF1 | ABCF2 | ABCF3 | ABCG1 |
| ABCG2 | ABCG4 | ABHD10 | ABHD11 | ABHD12 | ABHD13 | ABHD14A | ABHD14A-ACY1 |
| ABHD14B | ABHD15 | ABHD16A | ABHD16B | ABHD17A | ABHD17B | ABHD17C | ABHD18 |
| ABHD2 | ABHD3 | ABHD4 | ABHD5 | ABHD6 | ABHD8 | ABI1 | ABI2 |
| ABI3 | ABI3BP | ABL1 | ABL2 | ABLIM1 | ABLIM3 | ABO | ABR |
| ABRACL | ABT1 | ABTB1 | ABTB2 | AC002310.11 | AC002310.13 | AC002398.9 | AC002985.3 |
| AC003002.4 | AC003002.6 | AC003005.4 | AC003006.7 | AC004076.7 | AC004076.9 | AC004158.1 | AC004754.3 |
| AC005779.2 | AC005943.2 | AC006026.1 | AC006445.1 | AC006449.2 | AC006486.9 | AC006538.4 | AC007040.11 |
| AC007192.4 | AC007326.1 | AC007906.1 | AC008074.1 | AC008522.1 | AC008641.1 | AC008759.1 | AC009022.1 |
| AC009065.1 | AC009121.1 | AC009133.22 | AC009133.23 | AC009336.19 | AC009950.1 | AC010547.9 | AC010642.1 |
| AC010646.3 | AC011380.1 | AC011513.3 | AC011530.4 | AC013264.1 | AC013461.1 | AC015688.3 | AC016549.1 |
| AC017028.1 | AC018638.1 | AC018755.18 | AC021106.1 | AC023283.1 | AC023908.1 | AC024060.1 | AC024361.1 |
| AC024592.12 | AC025263.3 | AC026348.1 | AC027682.1 |  |  |  |  |

URL for the ERG gene set/The exoRbase: <http://exorbase.org/database/>.

**Supplementary Table S4.** The GO descriptions details for differential expressed ERGs.

| **GO** | **ID** | **Description** | **BgRatio** | **p.adjust** | **qvalue** |
| --- | --- | --- | --- | --- | --- |
| BP | GO:0035640 | exploration behavior | 26/18670 | 0.003 | 0.001 |
| BP | GO:0035023 | regulation of Rho protein signal transduction | 140/18670 | 0.033 | 0.011 |
| BP | GO:0050866 | negative regulation of cell activation | 199/18670 | 0.033 | 0.011 |
| BP | GO:0007266 | Rho protein signal transduction | 203/18670 | 0.033 | 0.011 |
| BP | GO:1903531 | negative regulation of secretion by cell | 211/18670 | 0.033 | 0.011 |
| BP | GO:0046578 | regulation of Ras protein signal transduction | 238/18670 | 0.033 | 0.011 |
| BP | GO:0051048 | negative regulation of secretion | 238/18670 | 0.033 | 0.011 |
| BP | GO:0030100 | regulation of endocytosis | 281/18670 | 0.033 | 0.011 |
| BP | GO:0051056 | regulation of small GTPase mediated signal transduction | 338/18670 | 0.033 | 0.011 |
| BP | GO:0070472 | regulation of uterine smooth muscle contraction | 10/18670 | 0.033 | 0.011 |
| BP | GO:0032102 | negative regulation of response to external stimulus | 365/18670 | 0.033 | 0.011 |
| BP | GO:0002887 | negative regulation of myeloid leukocyte mediated immunity | 11/18670 | 0.033 | 0.011 |
| BP | GO:0031652 | positive regulation of heat generation | 11/18670 | 0.033 | 0.011 |
| BP | GO:0070471 | uterine smooth muscle contraction | 11/18670 | 0.033 | 0.011 |
| BP | GO:1902563 | regulation of neutrophil activation | 11/18670 | 0.033 | 0.011 |
| BP | GO:0051956 | negative regulation of amino acid transport | 12/18670 | 0.033 | 0.011 |
| BP | GO:0090331 | negative regulation of platelet aggregation | 12/18670 | 0.033 | 0.011 |
| BP | GO:0015812 | gamma-aminobutyric acid transport | 13/18670 | 0.033 | 0.011 |
| BP | GO:0031650 | regulation of heat generation | 13/18670 | 0.033 | 0.011 |
| BP | GO:0043301 | negative regulation of leukocyte degranulation | 13/18670 | 0.033 | 0.011 |
| BP | GO:0060312 | regulation of blood vessel remodeling | 13/18670 | 0.033 | 0.011 |
| BP | GO:0050804 | modulation of chemical synaptic transmission | 436/18670 | 0.033 | 0.011 |
| BP | GO:0099177 | regulation of trans-synaptic signaling | 437/18670 | 0.033 | 0.011 |
| BP | GO:0033604 | negative regulation of catecholamine secretion | 15/18670 | 0.033 | 0.011 |
| BP | GO:0034111 | negative regulation of homotypic cell-cell adhesion | 15/18670 | 0.033 | 0.011 |
| BP | GO:0007265 | Ras protein signal transduction | 448/18670 | 0.033 | 0.011 |
| BP | GO:0031649 | heat generation | 17/18670 | 0.033 | 0.011 |
| BP | GO:0060080 | inhibitory postsynaptic potential | 17/18670 | 0.033 | 0.011 |
| BP | GO:0051957 | positive regulation of amino acid transport | 18/18670 | 0.033 | 0.011 |
| BP | GO:0090330 | regulation of platelet aggregation | 18/18670 | 0.033 | 0.011 |
| BP | GO:0010544 | negative regulation of platelet activation | 19/18670 | 0.033 | 0.011 |
| BP | GO:0007620 | copulation | 20/18670 | 0.033 | 0.011 |
| BP | GO:0034104 | negative regulation of tissue remodeling | 20/18670 | 0.033 | 0.011 |
| BP | GO:0042053 | regulation of dopamine metabolic process | 20/18670 | 0.033 | 0.011 |
| BP | GO:0042069 | regulation of catecholamine metabolic process | 20/18670 | 0.033 | 0.011 |
| BP | GO:0048148 | behavioral response to cocaine | 20/18670 | 0.033 | 0.011 |
| BP | GO:0032891 | negative regulation of organic acid transport | 21/18670 | 0.033 | 0.011 |
| BP | GO:0042135 | neurotransmitter catabolic process | 21/18670 | 0.033 | 0.011 |
| BP | GO:0035024 | negative regulation of Rho protein signal transduction | 22/18670 | 0.033 | 0.011 |
| BP | GO:0015740 | C4-dicarboxylate transport | 23/18670 | 0.033 | 0.011 |
| BP | GO:0051589 | negative regulation of neurotransmitter transport | 23/18670 | 0.033 | 0.011 |
| BP | GO:0034110 | regulation of homotypic cell-cell adhesion | 25/18670 | 0.034 | 0.012 |
| BP | GO:1903306 | negative regulation of regulated secretory pathway | 25/18670 | 0.034 | 0.012 |
| BP | GO:0051953 | negative regulation of amine transport | 27/18670 | 0.036 | 0.012 |
| BP | GO:0002691 | regulation of cellular extravasation | 28/18670 | 0.036 | 0.012 |
| BP | GO:1903792 | negative regulation of anion transport | 28/18670 | 0.036 | 0.012 |
| BP | GO:0010543 | regulation of platelet activation | 31/18670 | 0.038 | 0.013 |
| BP | GO:0045987 | positive regulation of smooth muscle contraction | 32/18670 | 0.038 | 0.013 |
| BP | GO:0010039 | response to iron ion | 33/18670 | 0.038 | 0.013 |
| BP | GO:0051955 | regulation of amino acid transport | 33/18670 | 0.038 | 0.013 |
| BP | GO:2001024 | negative regulation of response to drug | 33/18670 | 0.038 | 0.013 |
| BP | GO:0032892 | positive regulation of organic acid transport | 34/18670 | 0.038 | 0.013 |
| BP | GO:0045920 | negative regulation of exocytosis | 34/18670 | 0.038 | 0.013 |
| BP | GO:0051954 | positive regulation of amine transport | 35/18670 | 0.038 | 0.013 |
| BP | GO:0038083 | peptidyl-tyrosine autophosphorylation | 37/18670 | 0.038 | 0.013 |
| BP | GO:0043114 | regulation of vascular permeability | 37/18670 | 0.038 | 0.013 |
| BP | GO:2000249 | regulation of actin cytoskeleton reorganization | 37/18670 | 0.038 | 0.013 |
| BP | GO:0042417 | dopamine metabolic process | 39/18670 | 0.039 | 0.013 |
| BP | GO:0007618 | mating | 40/18670 | 0.039 | 0.013 |
| BP | GO:0014046 | dopamine secretion | 40/18670 | 0.039 | 0.013 |
| BP | GO:0014059 | regulation of dopamine secretion | 40/18670 | 0.039 | 0.013 |
| BP | GO:0010863 | positive regulation of phospholipase C activity | 43/18670 | 0.04 | 0.014 |
| BP | GO:0001974 | blood vessel remodeling | 45/18670 | 0.04 | 0.014 |
| BP | GO:1900274 | regulation of phospholipase C activity | 45/18670 | 0.04 | 0.014 |
| BP | GO:0043300 | regulation of leukocyte degranulation | 46/18670 | 0.04 | 0.014 |
| BP | GO:0045776 | negative regulation of blood pressure | 46/18670 | 0.04 | 0.014 |
| BP | GO:0045933 | positive regulation of muscle contraction | 48/18670 | 0.04 | 0.014 |
| BP | GO:0002686 | negative regulation of leukocyte migration | 49/18670 | 0.04 | 0.014 |
| BP | GO:0002704 | negative regulation of leukocyte mediated immunity | 49/18670 | 0.04 | 0.014 |
| BP | GO:0035094 | response to nicotine | 49/18670 | 0.04 | 0.014 |
| BP | GO:1903793 | positive regulation of anion transport | 50/18670 | 0.04 | 0.014 |
| BP | GO:0015872 | dopamine transport | 52/18670 | 0.04 | 0.014 |
| BP | GO:0046580 | negative regulation of Ras protein signal transduction | 52/18670 | 0.04 | 0.014 |
| BP | GO:0050885 | neuromuscular process controlling balance | 52/18670 | 0.04 | 0.014 |
| BP | GO:0030195 | negative regulation of blood coagulation | 53/18670 | 0.04 | 0.014 |
| BP | GO:0006584 | catecholamine metabolic process | 54/18670 | 0.04 | 0.014 |
| BP | GO:0009712 | catechol-containing compound metabolic process | 54/18670 | 0.04 | 0.014 |
| BP | GO:0051353 | positive regulation of oxidoreductase activity | 54/18670 | 0.04 | 0.014 |
| BP | GO:1900047 | negative regulation of hemostasis | 54/18670 | 0.04 | 0.014 |
| BP | GO:0002886 | regulation of myeloid leukocyte mediated immunity | 55/18670 | 0.04 | 0.014 |
| BP | GO:0042220 | response to cocaine | 55/18670 | 0.04 | 0.014 |
| BP | GO:0050819 | negative regulation of coagulation | 57/18670 | 0.04 | 0.014 |
| BP | GO:0051058 | negative regulation of small GTPase mediated signal transduction | 58/18670 | 0.04 | 0.014 |
| BP | GO:0010518 | positive regulation of phospholipase activity | 59/18670 | 0.04 | 0.014 |
| BP | GO:0032890 | regulation of organic acid transport | 59/18670 | 0.04 | 0.014 |
| BP | GO:0070527 | platelet aggregation | 59/18670 | 0.04 | 0.014 |
| BP | GO:0045123 | cellular extravasation | 61/18670 | 0.041 | 0.014 |
| BP | GO:0050433 | regulation of catecholamine secretion | 62/18670 | 0.041 | 0.014 |
| BP | GO:0031646 | positive regulation of neurological system process | 63/18670 | 0.041 | 0.014 |
| BP | GO:0050432 | catecholamine secretion | 64/18670 | 0.042 | 0.014 |
| BP | GO:0006940 | regulation of smooth muscle contraction | 65/18670 | 0.042 | 0.014 |
| BP | GO:0015800 | acidic amino acid transport | 66/18670 | 0.042 | 0.014 |
| BP | GO:0071300 | cellular response to retinoic acid | 69/18670 | 0.043 | 0.015 |
| BP | GO:0010517 | regulation of phospholipase activity | 70/18670 | 0.043 | 0.015 |
| BP | GO:0050766 | positive regulation of phagocytosis | 70/18670 | 0.043 | 0.015 |
| BP | GO:0042273 | ribosomal large subunit biogenesis | 71/18670 | 0.043 | 0.015 |
| BP | GO:0032024 | positive regulation of insulin secretion | 72/18670 | 0.043 | 0.015 |
| BP | GO:0060193 | positive regulation of lipase activity | 72/18670 | 0.043 | 0.015 |
| BP | GO:0061045 | negative regulation of wound healing | 76/18670 | 0.045 | 0.015 |
| BP | GO:0051937 | catecholamine transport | 77/18670 | 0.045 | 0.016 |
| BP | GO:0072347 | response to anesthetic | 78/18670 | 0.045 | 0.016 |
| BP | GO:0030193 | regulation of blood coagulation | 79/18670 | 0.045 | 0.016 |
| BP | GO:1900046 | regulation of hemostasis | 80/18670 | 0.045 | 0.016 |
| BP | GO:0033238 | regulation of cellular amine metabolic process | 81/18670 | 0.045 | 0.016 |
| BP | GO:0034109 | homotypic cell-cell adhesion | 81/18670 | 0.045 | 0.016 |
| BP | GO:0008652 | cellular amino acid biosynthetic process | 83/18670 | 0.046 | 0.016 |
| BP | GO:0050818 | regulation of coagulation | 84/18670 | 0.046 | 0.016 |
| BP | GO:0034103 | regulation of tissue remodeling | 88/18670 | 0.048 | 0.016 |
| BP | GO:0015844 | monoamine transport | 90/18670 | 0.048 | 0.016 |
| BP | GO:1903035 | negative regulation of response to wounding | 90/18670 | 0.048 | 0.016 |
| BP | GO:0014909 | smooth muscle cell migration | 91/18670 | 0.048 | 0.017 |
| BP | GO:0044070 | regulation of anion transport | 95/18670 | 0.048 | 0.017 |
| BP | GO:0051952 | regulation of amine transport | 95/18670 | 0.048 | 0.017 |
| BP | GO:0006835 | dicarboxylic acid transport | 96/18670 | 0.048 | 0.017 |
| BP | GO:0090277 | positive regulation of peptide hormone secretion | 96/18670 | 0.048 | 0.017 |
| BP | GO:0090630 | activation of GTPase activity | 97/18670 | 0.048 | 0.017 |
| BP | GO:0042472 | inner ear morphogenesis | 98/18670 | 0.048 | 0.017 |
| BP | GO:0050764 | regulation of phagocytosis | 98/18670 | 0.048 | 0.017 |
| BP | GO:0060191 | regulation of lipase activity | 98/18670 | 0.048 | 0.017 |
| BP | GO:2001023 | regulation of response to drug | 100/18670 | 0.048 | 0.017 |
| BP | GO:0031532 | actin cytoskeleton reorganization | 101/18670 | 0.048 | 0.017 |
| BP | GO:0015837 | amine transport | 102/18670 | 0.048 | 0.017 |
| BP | GO:0021549 | cerebellum development | 102/18670 | 0.048 | 0.017 |
| BP | GO:0018958 | phenol-containing compound metabolic process | 103/18670 | 0.048 | 0.017 |
| BP | GO:0014812 | muscle cell migration | 104/18670 | 0.048 | 0.017 |
| BP | GO:0042136 | neurotransmitter biosynthetic process | 106/18670 | 0.048 | 0.017 |
| BP | GO:0050905 | neuromuscular process | 107/18670 | 0.048 | 0.017 |
| BP | GO:0051341 | regulation of oxidoreductase activity | 107/18670 | 0.048 | 0.017 |
| BP | GO:0015696 | ammonium transport | 108/18670 | 0.048 | 0.017 |
| BP | GO:0032526 | response to retinoic acid | 108/18670 | 0.048 | 0.017 |
| BP | GO:0099565 | chemical synaptic transmission, postsynaptic | 108/18670 | 0.048 | 0.017 |
| BP | GO:0006939 | smooth muscle contraction | 110/18670 | 0.049 | 0.017 |
| BP | GO:0022037 | metencephalon development | 111/18670 | 0.049 | 0.017 |
| BP | GO:0043279 | response to alkaloid | 112/18670 | 0.049 | 0.017 |
| CC | GO:0099092 | postsynaptic density, intracellular component | 18/19717 | 0.021 | 0.013 |
| CC | GO:0099091 | postsynaptic specialization, intracellular component | 21/19717 | 0.021 | 0.013 |
| CC | GO:0045171 | intercellular bridge | 59/19717 | 0.04 | 0.025 |
| CC | GO:0098685 | Schaffer collateral - CA1 synapse | 82/19717 | 0.041 | 0.026 |
| MF | GO:0008483 | transaminase activity | 21/17697 | 0.04 | 0.012 |
| MF | GO:0016769 | transferase activity, transferring nitrogenous groups | 23/17697 | 0.04 | 0.012 |
| MF | GO:0003785 | actin monomer binding | 28/17697 | 0.04 | 0.012 |
| MF | GO:0001784 | phosphotyrosine residue binding | 40/17697 | 0.04 | 0.012 |
| MF | GO:0004715 | non-membrane spanning protein tyrosine kinase activity | 46/17697 | 0.04 | 0.012 |
| MF | GO:0045309 | protein phosphorylated amino acid binding | 51/17697 | 0.04 | 0.012 |
| MF | GO:0030170 | pyridoxal phosphate binding | 54/17697 | 0.04 | 0.012 |
| MF | GO:0070279 | vitamin B6 binding | 54/17697 | 0.04 | 0.012 |
| MF | GO:0030145 | manganese ion binding | 62/17697 | 0.04 | 0.012 |
| MF | GO:0051536 | iron-sulfur cluster binding | 63/17697 | 0.04 | 0.012 |
| MF | GO:0051540 | metal cluster binding | 63/17697 | 0.04 | 0.012 |
| MF | GO:0005089 | Rho guanyl-nucleotide exchange factor activity | 79/17697 | 0.044 | 0.014 |
| MF | GO:0051219 | phosphoprotein binding | 83/17697 | 0.044 | 0.014 |

**Supplementary Table S5.** The KEGG descriptions details for differential expressed ERGs.

| ID | Description | BgRatio | p.adjust | qvalue |
| --- | --- | --- | --- | --- |
| hsa00650 | Butanoate metabolism | 28/8096 | 0.023 | 0.005 |
| hsa00410 | beta-Alanine metabolism | 31/8096 | 0.023 | 0.005 |
| hsa00640 | Propanoate metabolism | 34/8096 | 0.023 | 0.005 |
| hsa00250 | Alanine, aspartate and glutamate metabolism | 37/8096 | 0.023 | 0.005 |
| hsa00280 | Valine, leucine and isoleucine degradation | 48/8096 | 0.024 | 0.005 |
| hsa05416 | Viral myocarditis | 60/8096 | 0.025 | 0.005 |
| hsa04012 | ErbB signaling pathway | 85/8096 | 0.027 | 0.006 |
| hsa04727 | GABAergic synapse | 89/8096 | 0.027 | 0.006 |

**Supplementary Table S6.** The Calculation results between ERGs and immune cells.

| **Immune Cells** | **ERGs** | **Correlation** | **pvalue** | **Regulation** |
| --- | --- | --- | --- | --- |
| B cells memory | AAMP | 0.725 | 0.002 | postive |
| Plasma cells | AAMP | -0.572 | 0.026 | negative |
| T cells CD8 | AAMP | 0.875 | <0.001 | postive |
| T cells CD4 naive | AAMP | 0.435 | 0.106 | postive |
| T cells CD4 memory resting | AAMP | -0.289 | 0.296 | negative |
| T cells CD4 memory activated | AAMP | 0.096 | 0.734 | postive |
| T cells follicular helper | AAMP | -0.782 | 0.001 | negative |
| T cells regulatory (Tregs) | AAMP | -0.255 | 0.359 | negative |
| T cells gamma delta | AAMP | -0.736 | 0.002 | negative |
| NK cells resting | AAMP | 0.57 | 0.027 | postive |
| NK cells activated | AAMP | -0.493 | 0.062 | negative |
| Monocytes | AAMP | 0.231 | 0.407 | postive |
| Macrophages M0 | AAMP | -0.517 | 0.048 | negative |
| Macrophages M2 | AAMP | 0.415 | 0.124 | postive |
| Dendritic cells activated | AAMP | -0.747 | 0.001 | negative |
| Mast cells resting | AAMP | -0.176 | 0.53 | negative |
| Mast cells activated | AAMP | -0.115 | 0.684 | negative |
| Eosinophils | AAMP | -0.361 | 0.186 | negative |
| Neutrophils | AAMP | 0.877 | <0.001 | postive |
| B cells memory | ABAT | -0.281 | 0.31 | negative |
| Plasma cells | ABAT | 0.354 | 0.196 | postive |
| T cells CD8 | ABAT | -0.773 | 0.001 | negative |
| T cells CD4 naive | ABAT | -0.129 | 0.646 | negative |
| T cells CD4 memory resting | ABAT | 0.257 | 0.355 | postive |
| T cells CD4 memory activated | ABAT | 0.341 | 0.213 | postive |
| T cells follicular helper | ABAT | 0.416 | 0.123 | postive |
| T cells regulatory (Tregs) | ABAT | 0.227 | 0.416 | postive |
| T cells gamma delta | ABAT | 0.431 | 0.109 | postive |
| NK cells resting | ABAT | -0.224 | 0.422 | negative |
| NK cells activated | ABAT | 0.087 | 0.758 | postive |
| Monocytes | ABAT | -0.025 | 0.929 | negative |
| Macrophages M0 | ABAT | 0.325 | 0.237 | postive |
| Macrophages M2 | ABAT | -0.632 | 0.012 | negative |
| Dendritic cells activated | ABAT | 0.565 | 0.028 | postive |
| Mast cells resting | ABAT | 0.143 | 0.611 | postive |
| Mast cells activated | ABAT | 0.081 | 0.774 | postive |
| Eosinophils | ABAT | 0.278 | 0.316 | postive |
| Neutrophils | ABAT | -0.648 | 0.009 | negative |

**Supplementary Table S7.** The results of ROC analysis in training and validation set.

|  | **AUC** | **AUC [95%CI]** | **Sensitivity (%)** | **Specificity (%)** | **p-value** |
| --- | --- | --- | --- | --- | --- |
| AAMP |  |  |  |  |  |
| Training | 0.832 | 0.686 to 0.977 | 66.7 | 94.7 | <0.001 |
| Validatoin | 0.762 | 0.613 to 0.910 | 72.2 | 80.6 | <0.001 |
| ABAT |  |  |  |  |  |
| Training | 0.814 | 0.669 to 0.959 | 80.0 | 78.9 | <0.001 |
| Validatoin | 0.720 | 0.574 to 0.866 | 72.2 | 67.7 | 0.003 |
| AAMP/ABAT |  |  |  |  |  |
| Training | 0.891 | 0.773 to 1.000 | 80.0 | 94.7 | <0.001 |
| Validatoin | 0.787 | 0.644 to 0.930 | 72.2 | 80.6 | <0.001 |

**Abbreviations:** AUC, Area Under Curve.
