# Supplementary material for: A crucial exosome-related gene pair (AAMP and ABAT) is associated with inflammatory cells in intervertebral disc degeneration
Source: Front Immunol. 2023 Apr 14;14:1160801. doi: 10.3389/fimmu.2023.1160801 (PMC10140513; doi:10.3389/fimmu.2023.1160801)
Supplement: Supplementary file 2 [file Table_2.docx]

**Table R1. The enriched phosphorylation related pathways of regulation with the ceRNA network.**

| **GO** | **ID** | **Description** | **BgRatio** | **p-value** | **p adjust** | **q-value** | **Gene** |
| --- | --- | --- | --- | --- | --- | --- | --- |
| MF | GO:0030170 | pyridoxal phosphate binding | 54/17697 | 0.0122 | 0.0399 | 0.0122 | ABAT |
| BP | GO:0038083 | peptidyl-tyrosine autophosphorylation | 37/18670 | 0.0079 | 0.0381 | 0.0131 | ABL2 |
| MF | GO:0001784 | phosphotyrosine residue binding | 40/17697 | 0.0090 | 0.0399 | 0.0122 | ABL2 |
| BP | GO:0010863 | positive regulation of phospholipase C activity | 43/18670 | 0.0092 | 0.0398 | 0.0137 | ABL2 |
| BP | GO:1900274 | regulation of phospholipase C activity | 45/18670 | 0.0096 | 0.0398 | 0.0137 | ABL2 |
| MF | GO:0045309 | protein phosphorylated amino acid binding | 51/17697 | 0.0115 | 0.0399 | 0.0122 | ABL2 |
| BP | GO:0010518 | positive regulation of phospholipase activity | 59/18670 | 0.0126 | 0.0402 | 0.0139 | ABL2 |
| BP | GO:0010517 | regulation of phospholipase activity | 70/18670 | 0.0149 | 0.0430 | 0.0148 | ABL2 |
| MF | GO:0051219 | phosphoprotein binding | 83/17697 | 0.0186 | 0.0444 | 0.0136 | ABL2 |

**Table R2. The miRNA expression, p-value, and predicted sources.**

| **DE miRNA** | **Gene** | **miRDB** | **miRTarBase** | **TargetScan** | **Sum** | **AveExpression** | **p-value** |
| --- | --- | --- | --- | --- | --- | --- | --- |
| hsa-miR-24-3p | AAMP | NA | Y | Y | 2 | 13.5376 | <0.0001 |
| hsa-miR-590-5p | ABAT | Y | NA | Y | 2 | 1.9364 | 0.0030 |
| hsa-miR-27a-3p | ABAT | Y | NA | Y | 2 | 10.2252 | 0.0002 |
